# Antiretroviral Therapy Improves Acquired Immunodeficiency Syndrome with Systemic Lupus Erythematosus

**DOI:** 10.3390/life11060463

**Published:** 2021-05-21

**Authors:** Akinori Okada, Yuji Nozaki, Shinya Rai, Koji Kinoshita, Masanori Funauchi, Itaru Matsumura

**Affiliations:** Department of Hematology and Rheumatology, Faculty of Medicine, Kindai University, Osaka-Sayama, Osaka 589-8511, Japan; 161896@med.kindai.ac.jp (A.O.); rai@med.kindai.ac.jp (S.R.); kkino@med.kindai.ac.jp (K.K.); mn-funa@med.kindai.ac.jp (M.F.); imatsumura@med.kindai.ac.jp (I.M.)

**Keywords:** acquired immunodeficiency syndrome, systemic lupus erythematosus, human immunodeficiency virus, antiretroviral therapy, autoimmune disease

## Abstract

A 35-year-old male was referred to our hospital with dysesthesia of the lower extremities that had begun six months earlier. A blood test revealed the presence of various antibodies, suggesting a collagen-related peripheral neuropathy. However, a history of repeated shingles and sex with males was noted, and the patient was tested for and diagnosed with human immunodeficiency virus (HIV) infection. Based on the manifestations and laboratory data, including the results of immunological and urinary tests, he was further diagnosed with concomitant systemic lupus erythematosus (SLE). The activity of SLE improved with antiretroviral therapy. There is currently no established treatment for AIDS complicated with SLE. Indeed, because HIV treatment involves the activation of immune function and SLE treatment involves immunosuppression, any treatments for the two conditions would be in conflict. It is thus necessary to select a treatment strategy based on the condition of the individual patient. In addition, because HIV infection is relatively rare in Japan compared to other countries, rheumatologists in Japan must keep HIV infection in mind as a differential diagnosis for autoimmune diseases.

## 1. Introduction

Human immunodeficiency virus (HIV) infection is a major global public health problem. According to the Joint United Nations Programme on HIV and AIDS (UNAIDS), an estimated 37.9 million people were living with HIV in 2018, and the global HIV infection rate among adults was about 0.8% at that time. Although it is common in sub-Saharan Africa, there are a small number of new cases of HIV infection in developed countries as a result of sexual activity and the reuse of syringes. Due to antiretroviral therapy (ART), the number of deaths from HIV infection is decreasing. However, with ART, even if the viral load (VL) remains below the detection sensitivity for several years, once the medication is discontinued, HIV infection will be exacerbated as a result of an increase in the VL, therefore long-term treatment remains a challenge. Systemic lupus erythematosus (SLE) is a chronic autoimmune disease that causes system inflammation and tissue damage throughout the body. Whereas the mechanism of reduced immune function in HIV infection is known to involve a decrease in CD4-positive T cells, the cause of immune function deterioration in SLE has not been adequately clarified, although a combination of genetic background and environmental factors are known to be involved. Specifically, it has been established that neutrophils and apoptotic cells supply antigens that trigger the onset of SLE and promote the expression of type I IFN from plasmacytoid dendritic cells (pDCs) [1]. In addition, autoreactive T cells produce interleukin (IL)-17, which activates neutrophils to infiltrate organs, and autoreactive B cells produce autoantibodies. In consideration of the above, it is theoretically expected that SLE activity is reduced during the active phase of HIV due to general immune dysfunction, including the dysfunction of pDC, and the results of several reports appear to confirm this [2,3]. However, there are only a small number of reports of HIV infection and SLE comorbidity, and the relationship between the two conditions is not well known. Several hypotheses have been put forward to explain the mechanism of autoimmune disease in HIV-infected patients, including a direct role of viral particles, immune complex-mediated diseases, dysregulation of the B/T lymphocyte interaction, molecular mimicry, and polyclonal B lymphocyte activation that might favor the synthesis of autoantibodies [4]. It has been pointed out that the effect of ART on HIV infection may cause autoimmune diseases such as immune reconstitution inflammatory syndrome (IRIS) due to over-immunity against both external and auto-antigens, and that the regulation of immune activation by ART may reduce the production of autoantibodies. The causal relationship between ART and autoimmune disease also remains unclear. We report here the case of a patient with SLE complicated by HIV and acquired immunodeficiency syndrome (AIDS), in whom both conditions improved with ART.

## 2. Case Presentation

A 35-year-old male who was generally healthy began to experience abnormal sensations in the left sole of foot and myalgia in the gastrocnemius. He also had recurring fever and shingles over 39 degrees. After experiencing these symptoms intermittently for 6 months, he consulted his regular physician, who performed a blood test, peripheral nerve conduction velocity test, and head MRI (magnetic resonance imaging). Although there were no abnormal findings on the head MRI, the blood test was positive for anti-SS-A, anti-SS-B, and anti-Sm antibodies, and the peripheral nerve conduction velocity test revealed peripheral nerve conduction defects in the gastrocnemius. He was referred to our hospital. Physical examination showed no abnormalities in the central nervous system, but revealed hypoesthesia of the lower extremities, predominantly on the left. The results of the laboratory investigation are shown in Table 1. A blood test showed negative antinuclear antibody (ANA), positive anti-ds-DNA antibody, positive anti-Sm antibody, hypergammaglobulinemia, hypocomplementemia, and estimated daily urine protein of 1.0 g/day, suggesting the possibility of neurological symptoms associated with collagen disease.

**Figure 1:**
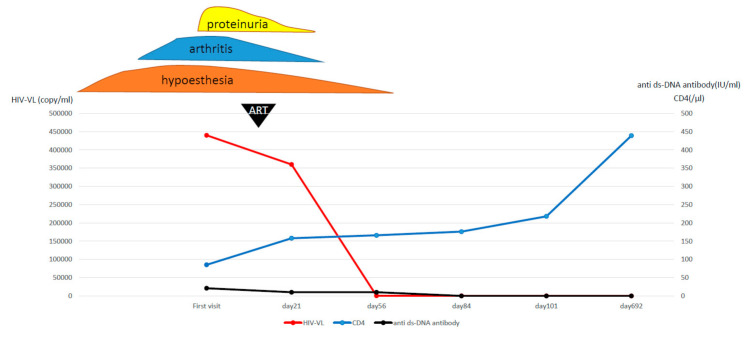
The clinical course of the patients.

He also had a history of multiple viral infections, showed signs suspicious of HIV infection, and tested positive for HIV antibodies, which were also positive on HIV–RNA PCR and Western blot assays. He was diagnosed with AIDS complicated by HIV encephalopathy based on a CD4-positive T cell count of 86/µL in peripheral blood, an elevated cerebrospinal fluid (CSF) cell count and protein in the CSF. Both the American College of Rheumatology criteria and the Systemic Lupus International Collaborating Clinic criteria were met, therefore we diagnosed him with AIDS and SLE. The differential diagnosis was neuropsychiatric SLE, but because his CD4-positive T cell count was very low and the risk of infection was also high, we decided to prioritize the treatment of AIDS and start ART. Upon detailed questioning, it was revealed that he had had sex with males in his 20s, which was thought to be the cause of his HIV infection. Two months after the start of ART, HIV VL, which was 4.4 × 10^5^ copies/mL at the start of treatment, decreased to below detection sensitivity, the levels of anti-ds-DNA antibody and complement normalized, and the disease activity of SLE decreased. His joint symptoms disappeared and the numbness in both lower limbs improved, albeit mildly. In addition, laboratory findings showed an improvement in inflammatory findings and daily urine protein to 0.17 g/day. The patient was discharged from our hospital without any complications associated with ART. No therapeutic intervention has been given for SLE since then, but he has remained asymptomatic at follow-up. The clinical course of the patient is shown in Figure 1.

## 3. Discussion

Nearly 30 years after Kopelman et al. first reported a case of SLE complicated by HIV infection [5], the relationship between the etiology of HIV infection and SLE remains unclear, and there remain few reports of concomitant SLE and HIV worldwide. In 1993, Barthel et al. stated that based on epidemiological data for HIV and SLE, the number of patients with complications should be as high as 400 in the United States alone [6]. To date, however, only about 80 cases of SLE and HIV have been reported worldwide, and the relationship between these diseases is unclear [7,8]. We consider that there are several possible reasons for the small number of reports. Firstly, nearly 60% of all HIV patients are in Africa. Many countries here are developing and some patients are unable to be seen at hospitals due to economic reasons; therefore, the exact transmission to the number of persons is expected to be masked. Another reason is that HIV infection is mainly transmitted by male homosexuals in Japan, and thus comorbidity with SLE, which is more common in women, is very rare. Secondly, IL-16 is known to be high in SLE patients and correlates with disease activity, and IL-16 has been shown to inhibit HIV replication at the transcriptional level in vitro and to function defensively against HIV infection [9,10]. Thirdly, SLE may also function prophylactically in HIV infection as a result of polyclonal antibody production, and it has been reported that hydroxychloroquine, a therapeutic agent of SLE, inhibits HIV-1 transcription in T cells [11]. In addition, because T cells induce local inflammation through cytokine production and contribute to tissue damage, the depletion of CD4-positive T cells may block the disappearance of local inflammation and the production of autoantibodies in B cells [12].

The patient in this report had SLE and AIDS that developed about 10 years after a period of sexual activity with males. He was probably infected with HIV prior to his visit to our hospital and had been asymptomatic for some time, indicating that he developed SLE following HIV infection. The positive rate of immunological antibodies (e.g., ANA, anti-DNA, and anti-RNP antibodies) in HIV-infected patients has been reported to be 44–95% [13], and a wide variety of autoantibodies have been found. Although the immunological mechanism of antibody production in HIV-infected patients is not clear, Laura et al. have hypothesized that the polyclonal proliferation of B cells promotes antibody production, and that molecular mimicry between HIV proteins and autoantigens triggers antibody cross-reactions [4]. Various findings on the mechanism of autoimmune disease-like symptoms in HIV patients have been reported, including that the HIV p17 protein produces autoantibodies to endogenous erythropoietin by molecular mimicry, causing autoimmune anemia [14], and that the HIV protein Nef produces antibodies to platelet GP IIIa, causing autoimmune thrombocytopenia [15]. Other mechanisms, such as increased platelet destruction by immune complexes in the early stages of HIV infection and decreased platelet production in the advanced stages due to viral infection of megakaryocytes, have also been identified [4]. It has also been pointed out that the HIV protein Tat may cross-react with CD40 in B cells to promote B cell activation and antibody production [16]. 

In 2017, there was an interesting report from Taiwan that compared two groups of HIV-infected patients, one receiving ART and another not receiving ART, which found that the patients who did not receive ART had a significantly higher incidence of Sjögren’s syndrome and SLE [17]. This could be the result of an exacerbated T cell imbalance, due to the lack of suppression of HIV, and not just the mechanism of molecular mimicry. SLE may also protect against the development of HIV infection by molecularly mimicking autoantibodies, which have been demonstrated to show molecular sequence homology between the autoantigens of SLE patients and the HIV proteins Gag and Env [18]. Antibody cross-reactivity has also been noted between a 70 kDa autoantigen, one of the epitopes of the RNP antigen, and the neutralizing epitope against the HIV envelope protein gp 120/41 [19]. 

In this case, after ART, proteinuria disappeared, joint symptoms improved, and antibody titers decreased. HIV-positive patients are at increased risk for both acute and chronic kidney disease. The classic kidney disease of HIV infection, HIV-associated nephropathy (HIVAN), has become less common with the widespread use of ART; however, there has been a simultaneous increase in the prevalence of other kidney diseases. HIV-positive patients are also exposed to lifelong ART, with the potential to cause or exacerbate kidney injury. The spectrum of renal pathology in HIV-positive patients is diverse, including lesions directly related to intrarenal HIV gene expression and lesions related to comorbidities, drug effects, immune dysregulation, and co-infections [20]. The main pathologic classification of HIVANs divides them into two types, glomerular and tubulointerstitial dominant. The glomerular dominant HIVANs are further divided into podocytopathies and immune complex-mediated glomerular disease [20]. Renal biopsy is required to distinguish between these lesions. However, in this case, the patient did not provide consent for a renal biopsy. If renal biopsy had been performed, we anticipate that an immune complex-mediated glomerular lesion would have been histologically detected in the kidney tissues due to the treatment-induced reduction in antibodies. 

Sae described a patient who was admitted to hospital for fever, pancytopenia, and proteinuria, determined to have SLE after a renal biopsy, and treated with immunosuppressive therapy [21]. However, after repeated unnatural infections, HIV was suspected and diagnosed. This previous report and our present case indicate that screening for HIV should be performed in even slightly atypical cases of SLE, because HIV symptoms can be masked to some extent by immunosuppressive therapy, and the course can be fatal. Additionally, as clinicians, we should be aware that HIV infection and SLE follow a very similar course.

## 4. Conclusions

It should be noted that SLE and HIV infection can have a very similar course due to their immunological pathogenesis.

## Figures and Tables

**Table 1:** The results of the laboratory investigation.

Blood		Immunology	
WBC	3500/µL	C3	71 mg/dL
Lymph	28.3%	C4	10 mg/dL
CD4	8.6%	ANA	Negative
CD8	77.9%	Anti-ds-DNA antibody	21 IU/mL
RBC	303 × 10⁴/µL	Anti-SS-A antibody	Negative
HGB	10.0 g/dL	Anti-SS-B antibody	Negative
PLT	23.0 × 10⁴/μL	Anti-Sm antibody	113.3 U/mL
			
Serology		Infection	
CRP	0.711 mg/dL	HIV-VL	4.4 × 10⁵ copy/mL
TP	9.0 g/dL		
Alb	2.9 g/dL	Urinalysis	
BUN	19 mg/dL	Creatinine	120 mg/dL
Creatinine	0.58 mg/dL	protein	128 mg/dL
IgG	4343 mg/dL	Estimated urine protein	1.0 g/day
IgA	155 mg/dL		
IgM	811 mg/dL	Cerebrospinal fluid	
		Cell Counts	43 /µL
		TP	65 mg/dL
